# Mulching Effects on Nutrient Contents of Potato Foliage and Colorado Potato Beetle Fitness

**DOI:** 10.1002/pei3.70059

**Published:** 2025-06-05

**Authors:** Christiane Weiler, Simeon Leisch, Stephan Martin Junge, Maria Renate Finckh

**Affiliations:** ^1^ Organic Agricultural Sciences, Section Ecological Plant Protection University of Kassel Witzenhausen Germany

**Keywords:** Chrysomelidae, conservation biocontrol, *Leptinotarsa decemlineata*, organic agriculture, pest management, regenerative agriculture

## Abstract

Application of organic mulches has repeatedly been shown to reduce infestation with *Leptinotarsa decemlineata* (Say) (Coleoptera: Chrysomelidae), the Colorado potato beetle (CPB). In order to determine if the nutritional status of potatoes as affected by mulch could explain the mulch effects in potatoes against CPB, we determined potato leaf nutrient composition in unmulched control plots and plots mulched with grass‐clover or triticale‐vetch and assessed mulch effects on CPB damage and development in the field during 3 years and under controlled conditions. In mulched plots, foliar Mo, Cl, and K contents were consistently higher than those without mulch, and leaf damage by CPB was reduced significantly. In addition, increased B contents were associated with undamaged plant material, while higher Zn contents were associated with leaves damaged by CPB. Under controlled conditions, CPB fitness was not affected by mulch application. Overall, reduced CPB damage could not be clearly attributed to altered foliar nutrient contents due to mulching. It is thus more likely that CPB reductions in mulched systems are due to mechanisms other than an altered nutrient balance.

## Graphical abstract

Application of organic mulches to potato fields has been demonstrated to reduce infestation by *Leptinotarsa decemlineata* (Say), the Colorado potato beetle (CPB), but the underlying mechanism remains unclear. Contrary to expectations, the observed reduction in CPB damage in mulched plots did not correlate with altered foliar nutrient profiles, suggesting that other factors may be responsible for the observed effect.

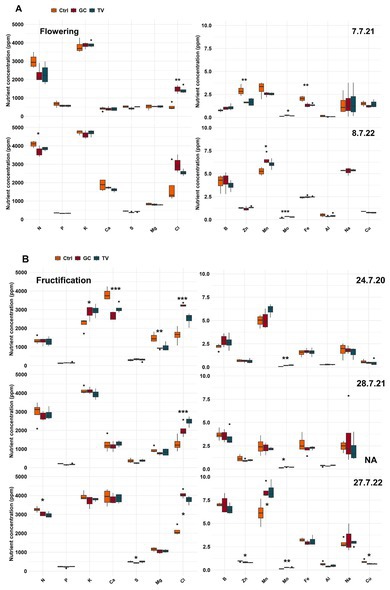

## 1 Introduction

Organic potato growers rely on preventive measures against Colorado potato beetle (CPB) *Leptinotarsa decemlineata* (Say) as well as the regular application of organically certified pesticides. Applying transferred organic mulches is an alternative agroecological approach for managing CPB and many other problems in potatoes (Junge and Finckh 2024). Although the effects of mulch on eggs, larvae, and adults of *L. decemlineata* have been described in several studies (Zehnder and Hough‐Goldstein 1990; Brust 1994; Stoner 1997; Szendrei et al. 2009; Junge et al. 2022; Winkler et al. 2024; Weiler et al. 2025, n.d.), the concrete underlying mechanisms have only been elucidated in part. Several mechanisms have been discussed to contribute to the reduction of CPB through mulching: (i) the mulching material could cause a disturbance of the *visual orientation* (Kirchner et al. 2014) or (ii) the *olfactory orientation* of CPB (Jermy et al. 1988); (iii) mulch (Szendrei et al. 2009; Weiler et al. n.d.) could also be a physical *barrier* to the spread of the CPB (Szendrei et al. 2009; Weiler et al. n.d.); (iv) mulch changes the microclimatic conditions: *insulation* provided by mulch leads to slower warming of the soil in spring and could delay the development of CPB. In addition, mulch may affect the microclimate within the plant canopy considerably due to its brighter albedo compared to soil and due to its hygroscopic properties. This leads to higher peak temperatures during the day that may damage eggs (Weiler et al. 2025); (v) Brust (1994) found significantly more *predators* of the CPB in mulched compared to unmulched potatoes; (vi) in a direct comparison of organic (cow manure with sawdust litter) and synthetically fertilized potatoes, higher boron and lower zinc concentrations in organically fertilized potato leaves were apparently related to a reduction of all developmental stages of the potato beetle (Alyokhin et al. 2005). In general, manure‐amended plots had different concentrations of nitrogen, calcium, magnesium, phosphorus, aluminum, boron, copper, iron, manganese, and zinc compared to the synthetically fertilized treatments, which explained 40%–57% of the variation in CPB populations in the field (Alyokhin et al. 2005). Alyokhin and Atlihan (2005) also observed reduced fecundity of female CPB, increased mortality of larval stage 1, reduced foliage consumption, and increased total developmental time of CPB on caged plants amended with manure compared to synthetic fertilizer. Alyokhin et al. (2005) deduced from this that the *mineral balance hypothesis*, first described by Phelan et al. (1996), could explain the effects on the potato‐CPB interaction.

Phelan et al. (1996) hypothesized that organic matter and microbial activity associated with organically managed soils can positively influence the nutrient balance of the plant and therefore promote plant growth and health. As synthetic fertilizers usually contain only certain nutrients such as nitrogen (N), phosphorus (P), or potassium (K), the nutrient balance with respect to most other nutrients in the plant may be affected. This may lead to an excess or lack of certain key nutrients for pests, supporting or suppressing their development, similar to the effects of mineral nutrients on plant pathogen development (Datnoff et al. 2023). With respect to CPB, the nutrient effects are not clear. While different levels of synthetic N fertilization (0, 56, 84, 140, and 224 kg/h) in potato did not affect the developmental time of CPB (Jansson and Smilowitz 1985), higher N concentration in potato foliage was correlated with higher CPB abundance and shorter developmental time (Jansson and Smilowitz 1986). Also, higher N fertilization in tomato resulted in shorter developmental time and increased survival (Hare and Dodds 1987; Hunt et al. 1992). Depending on the amount and kind of organic fertilizers applied, they may also promote CPB. For example, Boiteau et al. (2008) found that the excessive use (300 kg N/ha) of poultry manure as organic fertilizer reduced CPB larval development time by 22% compared to an unfertilized control. The picture is complicated by the fact that nutrient concentrations in the potato foliage develop dynamically with the initiation of tuber growth, with N, P, K, Cu, Zn, and S declining and Ca, Mg, B, Fe, Cl, and Mn increasing (Rowe and Westermann 1993). Errebhi et al. (1998) found that for optimal yields, potatoes needed 1300–1430 mg L^−1^ of NO_3_ during the tuber initiation stage, 550–1350 mg L^−1^ during tuber bulking, and 550–600 mg L^−1^ at maturity, assessed by plant sap analysis.

In the presented study, we evaluated the effect of mulching potatoes with transferred fresh organic materials on their foliar nutrient contents and the interaction of these with CPB infestation and development. The following questions were addressed: (i) Are there consistent effects of transferred organic mulch on the macro‐ and micronutrient contents of potato foliage? (ii) Are changes in foliar nutrient contents associated with the leaf damage caused by CPB and/or changes in CPB developmental time? For our investigation, we assessed CPB damage in field trials and, in parallel, took plant sap samples for nutrient analysis. As in the field migration, microclimate, and predation intermingle with potential effects of foliar nutrient contents on the development of CPB, we also conducted feeding trials with field‐collected leaves as well as controlled environment greenhouse trials with caged plants to determine (iii) if in the absence of migration, predation, and microclimatic differences effects on CPB development could be documented.

## 2 Materials and Methods

### 2.1 Field Site and Trial Set‐Up

The trials were conducted in 2020–2022 under central European conditions at the experimental farm of the University of Kassel in Neu‐Eichenberg (51°22′51″ N, 9°54′44″ E), North Hesse, Germany. The experimental field is located 223 m above sea level and has been managed organically since 1988. The average annual mean temperature for 1991–2020 was 9.3°C, and the average annual rainfall was 663 mm. The potato experiments 2020–2022 have been described in detail by Weiler et al. (2025). In brief, they were integrated in a 6‐year rotation with four replications (i.e., 24 main plots of 15 × 50 m) subject to non‐inversion tillage since 2015 on a haplic luvisol (see Figure S1). Pre‐crop was winter wheat, followed by a cover crop of triticale‐vetch. The potato cultivar “Laura” was planted with approximately 40,000 tubers ha^−1^ in 0.75 m dams on 22.04.2020, 03.05.2021, and 03.05.2022 (see Table S1). Plots were 4.5 m (six rows) wide and 12 m long. The outer rows (0.75 m) of each plot and two rows on either side of the main plots were left as edges. Unmulched control plots were fertilized with 100 kg N/ha with hair meal pellets, while mulched plots received ca 50 t/ha FM of freshly cut and chopped (around 5 cm) grass‐clover or triticale‐vetch mulch on 19.05.2020, 09.06.2021, and 25.05.2022, shortly before plant emergence. The C:N ratios of the applied mulches were between 21 and 43, resulting in 160–250 kg N/ha applied with the mulch (Henzel et al. 2025). For these C:N ratios, a potential inorganic N‐release between 50% and 0% during one growing season can be assumed (Sradnick and Feller 2020).

### 2.2 Field Assessments

The number of stems per potato plant was assessed on 29th of June 2020 (BBCH 65, flowering), 27th of July 2021, and 25th of July 2022 (BBCH 70, fruit development). The percentage of leaf damage due to CPB in the field was visually estimated repeatedly during the seasons (Table 1) on marked plants. In 2020–2022, the number of plants assessed per plot was 24, 30, and 16, respectively.

**TABLE 1:** On‐field assessments of plant sap sampling with developmental stage of potato (BBCH, numbers in brackets) and leaf damage of CPB, as well as listing of CPB experiments. Details on experimental design and assessments for the observational seasons 2020–2022. Mulch treatments were an unmulched control (Ctrl), grass‐clover (GC), and triticale‐vetch mulch (TV).

Assessment	2020	2021	2022	2023
Leaf nutrient composition	24.7. (81)	7.7. (60) 28.7. (70)	8.7. (66) 27.7. (70)	
Damaged versus undamaged leaves		13.7. (63)	8.7. (66)	12.7. (68)
CPB leaf damage on‐field	5 assessments (14.7.–24.7.)	10 assessments (22.6.–28.7.)	3 assessments (13.6.–26.7.)	
CPB greenhouse experiment		8.4.–3.6.		18.9.–2.11.
CPB feeding trial		7.7.–17.8.	16.6.–29.7.	

To determine macro‐ and micronutrients in the plant sap (Esteves et al. 2021), 30 young potato leaflets, each from a different plant, were sent as a bulk sample per plot for analysis to NovacropControl in the Netherlands. In 2020, samples were taken at the developmental stage BBCH 81 at potato fruit ripening; in 2021 and 2022, potatoes were sampled twice, once after the beginning and at the end of flowering (BBCH 60 and 70, respectively) (Table 1).

In order to determine if and how CPB damage is affected by mulching, we compared leaf nutrient contents in damaged and undamaged plants from plants mulched with triticale‐vetch or unmulched plants in 2021 and 2022 from the experiments described above. In addition, we sampled the potato cultivar Anuschka in 2023 in an experiment conducted in a neighboring field with the same mulch materials; however, the plots were plowed (Table 1). Each year, we selected plots with and without mulch application and sampled leaves at a single date, either undamaged or damaged by CPB. In 2021, we randomly selected six plants with damaged and undamaged leaves in each plot and collected a mixed sample for each damage level. To enhance contrast, in 2022 and 2023, we focused on sampling highly damaged leaves in comparison to undamaged leaves from undamaged plants, collecting a mixed sample of damaged and a mixed sample of undamaged leaves in each plot.

### 2.3 Greenhouse and Feeding Experiments

**FIGURE 1:**
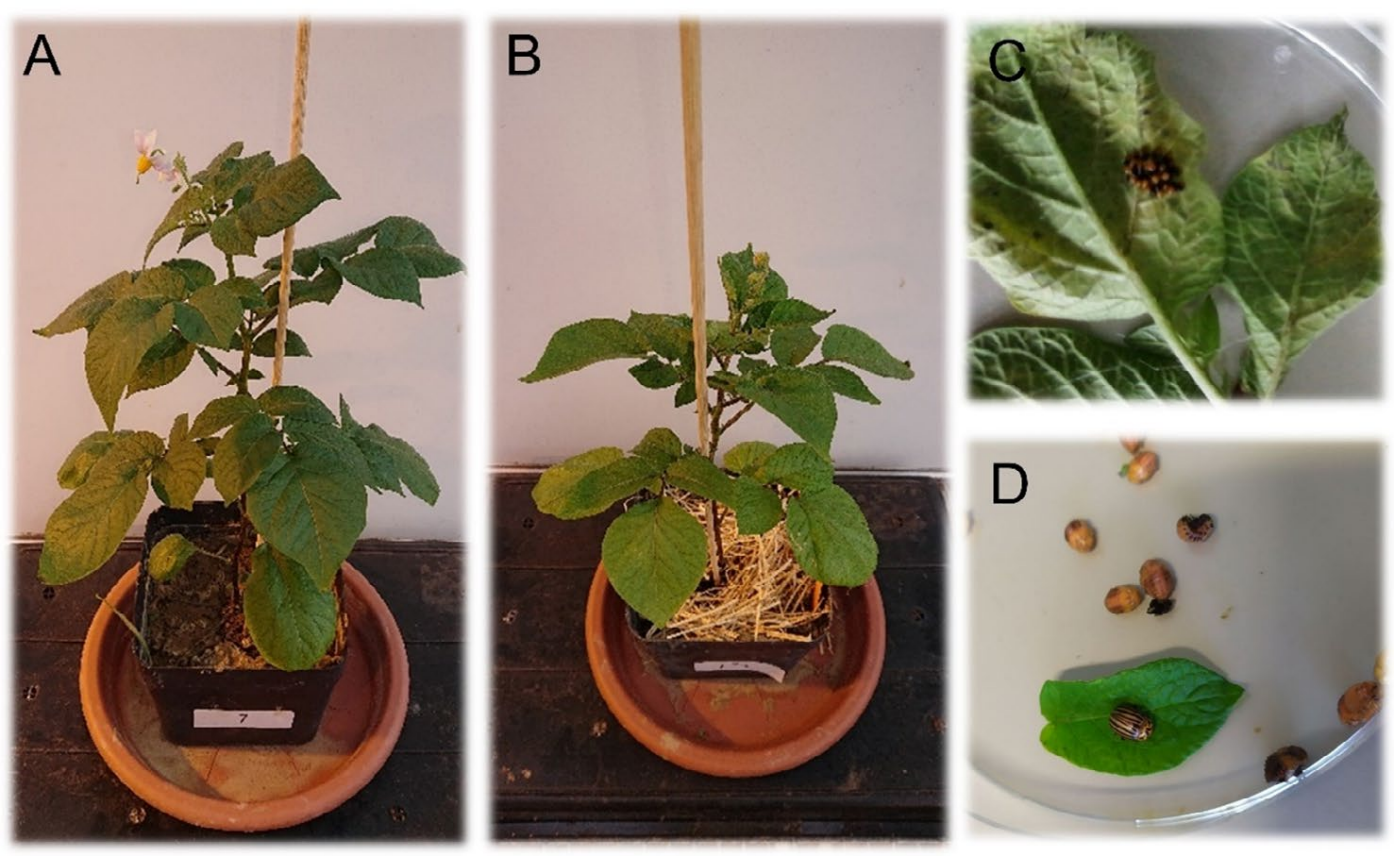
Unmulched (A) and mulched plants (B) used in greenhouse experiments. The plants were kept in 40 × 40 × 60 cages each. Petri dishes used in the feeding trials with recently hatched CPB eggs (C) and CPB pupae (D).

For the greenhouse experiments to assess CPB developmental dynamics under controlled conditions, excluding the influence of initial migration and migration, plants that had been fertilized with hair meal pellets (Figure 1A) were compared with plants mulched with hay (Figure 1B). In the first experiment, from 8.4. to 3.6.2021, mulch equivalent to 100 (M100) and 200 kg N/ha (M200) and hair meal pellets equivalent to 100 (100) and 200 kg N/ha (200) were applied, and each treatment (represented by one potted plant) was replicated four times. In the second experiment, from 18.9. to 2.11.2023, only 200 kg N/ha was applied, and both treatments (M200 and 200) were replicated 10 times. One couple of mature CPB from a colony reared in the greenhouse from a field collection was placed on each separately caged plant (cages were 40 × 40 × 60 cm), left until first oviposition, and then removed from the cage. The cages were kept at 24°C ± 2°C and 16:8 h photoperiod. In 2021, all larvae hatching from the egg mass were observed until the presence of adult CPB, while in 2023, during larval stage 1, individuals were reduced to 10 to better determine the duration of different developmental stages and ensure the availability of sufficient leaf material.

For feeding trials, four egg masses per plot were collected from the field on 06.07.21 (BBCH 59; before flowering) and 15.06.2022 (BBCH 25; side shoot development) from unmulched control plots (Ctrl) and plots with grass‐clover (GC) or triticale‐vetch mulch application (TV). Each treatment was replicated 4 times, resulting in 16 egg masses per treatment. Each egg mass was placed in an individual Petri dish and kept at 22°C and a 16:8 h photoperiod (Figure 1C). Throughout the experiment, larvae were fed with leaves from the plot they originated from to observe if potential nutritional changes caused by mulching would affect CPB development in the Petri dishes. Additionally, larval stage 4 larvae (last stage) were weighed daily in 2022.

### 2.4 Data Processing and Statistical Analysis

Data analyses were conducted using R 4.3.2. CPB population dynamics data have been published in detail by Weiler et al. (2025). Here, we concentrate on the damage caused by CPB. The area under the curve (AUC) (Jeger and Viljanen‐Rollinson 2001) was calculated for the CPB leaf damage per plot. Comparisons were performed with a Kruskal–Wallis test with a post hoc Dunn test, as data were not normally distributed (Table S2).

For the plant sap analysis, comparisons between the unmulched control (Ctrl), grass‐clover (GC), and triticale‐vetch (TV) mulch were performed with a Kruskal–Wallis test with Dunn test or ANOVA with Tukey test depending on the distribution of the data. Differences in nutrient contents of undamaged and damaged leaves from unmulched and mulched plots were analyzed and visualized with a redundancy analysis (Van Den Wollenberg 1977) (Table S2).

For the greenhouse experiments in 2021 and 2023, four models were compared using the AIC to determine the best model fit for each assessed life stage of CPB (eggs, larvae, and adults). The models compared were Poisson, negative binomial, zero‐inflated Poisson, and zero‐inflated negative binomial (Table S2). GLMs were calculated using MASS, pscl, and stats R packages. Pairwise comparisons were performed using a Tukey test with the emmeans R packages. Survival and hatching rates were compared by performing a Kruskal–Wallis test with a Dunn test, as data were not normally distributed (Table S2).

For the feeding experiments, the duration of each developmental stage of CPB was defined as the time between the first occurrence of one developmental stage (e.g., first individual in larval stage 2) until the molting of all individuals of this developmental stage to the next stage (e.g., all larval stage 2 larvae developed to the next stage), as several individuals were kept in one container, which can lead to overlapping durations and therefore an overestimated total duration. Total survival was calculated as the percentage of the original number compared to individuals reaching the adult stage. Significant differences were assessed by performing a Kruskal–Wallis test with a post hoc Dunn test, as data were not normally distributed (Table S2).

## 3 Results

The 2020 potato season (April to August) was comparatively cool with relatively dry conditions in April and July, while 2021 was characterized by a cool spring followed by rather warm temperatures with sufficient water and several heavy rainfall events. 2022 was extremely hot and dry, and 2023 was also relatively hot, but with a water surplus of 140 mm during the potato season (Table S3).

### 3.1 Leaf Damage

**FIGURE 2:**
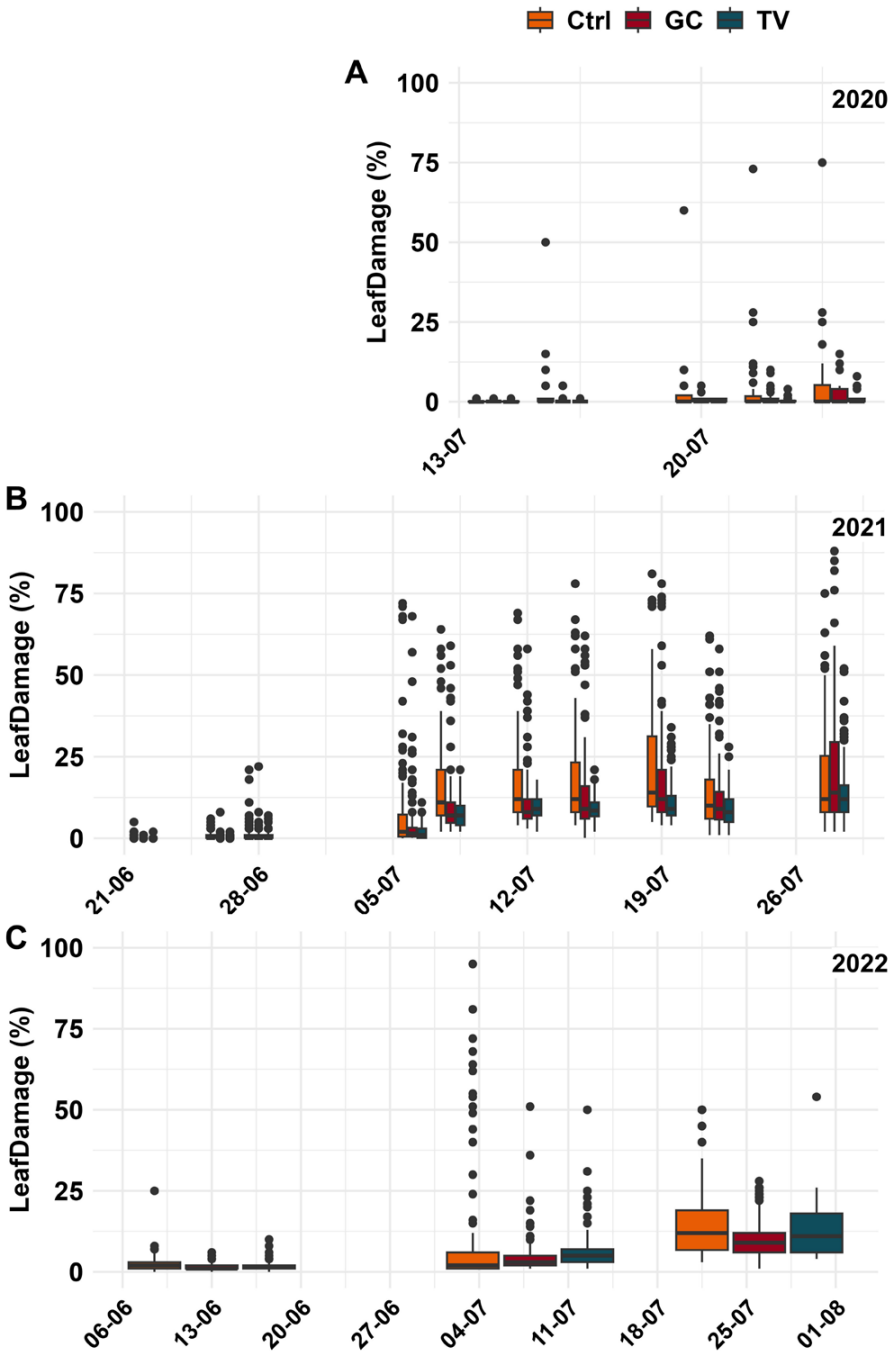
Boxplot for visually assessed leaf damage of CPB in the unmulched control (Ctrl) and plots with grass clover (GC) or triticale‐vetch (TV) mulch application for the observational years (A) 2020, (B) 2021 and (C) 2022. For significant differences of overall leaf damage per season, see Table S4.

The areas under the curves for leaf damage in 2020 were generally lower than in the other years due to the shorter observation period (Table 2). While damage in the unmulched plots was always highest, damage levels in the mulched plots varied. In 2021, damage was lowest in the plots mulched with triticale‐vetch, while in 2022, it was lowest when mulched with grass clover (Figure 2). The number of stems per potato plant, as a measurement of the agronomic performance of the plants, did not differ significantly (Table 2).

**TABLE 2:** Area under the curve (AUC) for visually assessed leaf damage in potato in the unmulched control (Ctrl) and plots with grass clover (GC) or triticale‐vetch (TV) mulch application and mean number of stems per potato plant.

Year	Treatment	AUC leaf damage (%)			Stems per plant	
		Mean	SE	*p*	Mean	SE
2020	Ctrl	22.8^a^	33.4	0.047	4.0	0.2
	GC	7.5^b^	6.4		3.7	0.3
	TV	4.4^b^	3.3		3.8	0.3
2021	Ctrl	418.1^a^	168.6	< 0.001	7.0	0.5
	GC	323.1^b^	120.2		7.1	0.2
	TV	222.2^c^	35.1		6.8	0.2
2022	Ctrl	370.5^ab^	240.3	< 0.001	4.7	0.2
	GC	212.1^b^	85.5		4.3	0.2
	TV	298.4^a^	114.1		4.1	0.3

*Note:* Significant differences were assessed via Kruskal–Wallis and Dunn tests within each year and are visualized with the letters a, b, and c. For sampling dates, see Table S1.

### 3.2 Macro‐ and Micronutrients in Leaf Material

**FIGURE 3:**
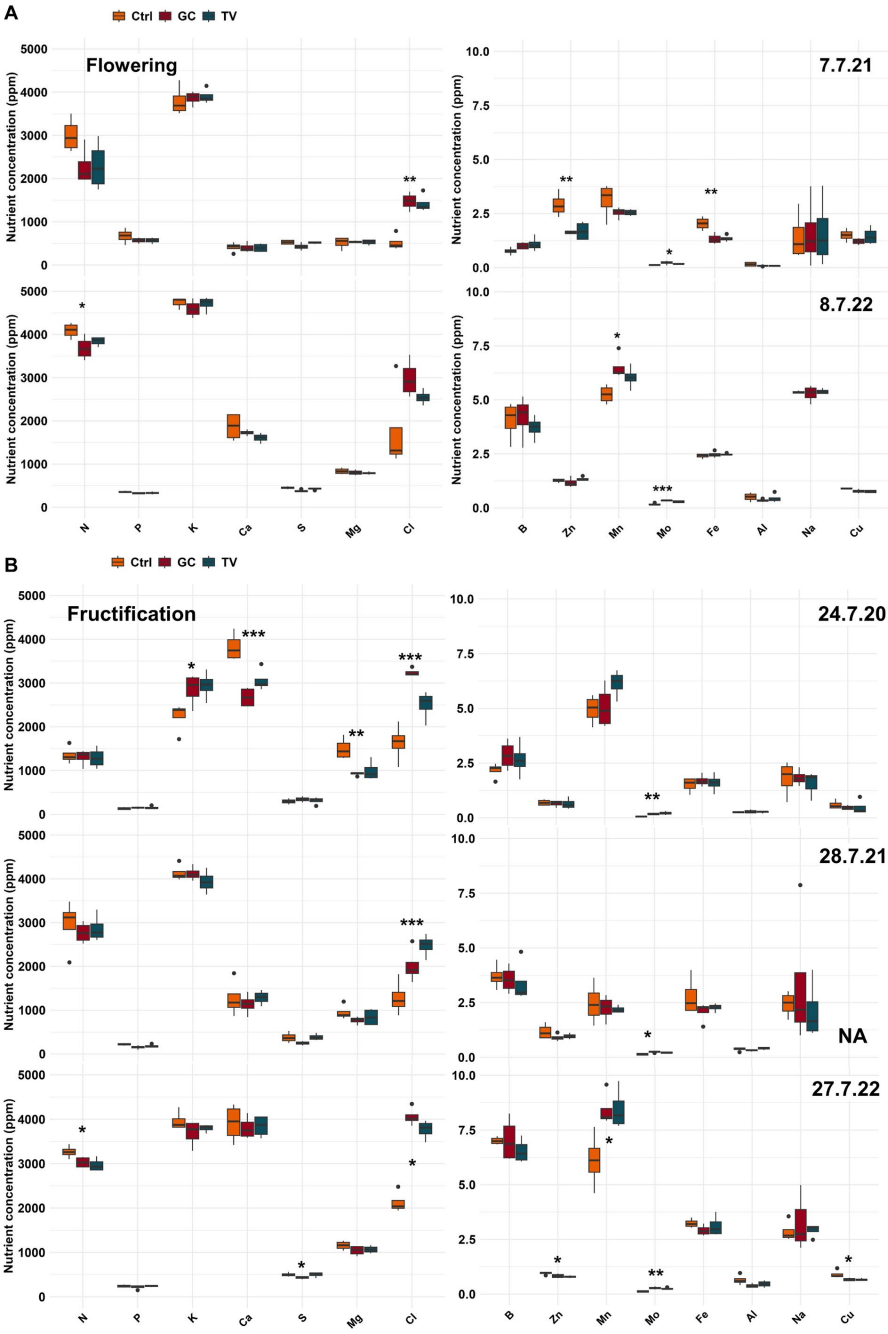
Macro‐ and micronutrient concentration for assessed nutrients (ppm) in the unmulched control (Ctrl) and plots with grass clover (GC) or triticale‐vetch (TV) mulch application for plant sap analysis on field at flowering (A) and fructification (B) of potato. Colors show different mulch treatments. For sampling dates, see Table 1. Differences among treatments with ***p < 0.001, **p < 0.01, and *p < 0.05 are indicated (Kruskal–Wallis with Dunn test or ANOVA with Tukey test). For reference values, see Table S4.

The foliar nutrient contents varied considerably depending on the nutrient and year. During the late July sampling done in all 3 years, N and K contents were lowest in 2020 compared to 2021 and 2022. In contrast, Ca levels were unusually low in 2021 compared to the other years, while B and Mn were highest in 2022 (Figure 3B).

In 2021 and 2022, when two samples were taken, differences in nutrient dynamics were apparent. Most conspicuous was the increase in the Cl levels over time, with a tendency for larger increases in the mulched treatments. The same was true for Mn in 2022 but not in 2021. Ca and B increased in both years, while Zn decreased. In contrast, N contents remained roughly the same over time in 2021 but fell in 2022 (Figure 3).

With the exception of Cl that was always significantly higher in the mulched treatments, effects of mulch on foliar macronutrient contents varied depending on year and sampling date. Among the macronutrients, N was mostly higher in the unmulched control treatments but differed only significantly in 2022 (Figure 3A,B, left). In mulched plots, K was significantly higher in 2020, when the general K levels were lower than in the two other years and below the reference values recommended by NovaCropControl (Table S4). Soil analyses also pointed to generally lower K‐levels in 2020 and increased K‐levels in the mulched variants (Table S5). In contrast to K, Ca and Mg were higher in the unmulched control (Figure 3), with no relevant variation in Mg between years or treatments in soil analyses (Table S5). Among the micronutrients, there was a trend towards lower Zn in mulched treatments: In 2021, Zn concentration was significantly lower in mulched plots only during flowering and in 2022 only during fructification (Figure 3, right). Only in the very hot and dry year 2022, Mn contents were significantly higher in the mulched treatments. Mo concentrations were always significantly higher in the mulched plots. Cu was slightly higher in the unmulched control with significant differences at fructification in 2022; the rest of the micronutrients did not show consistent trends or significant differences (Figure 3).

**FIGURE 4:**
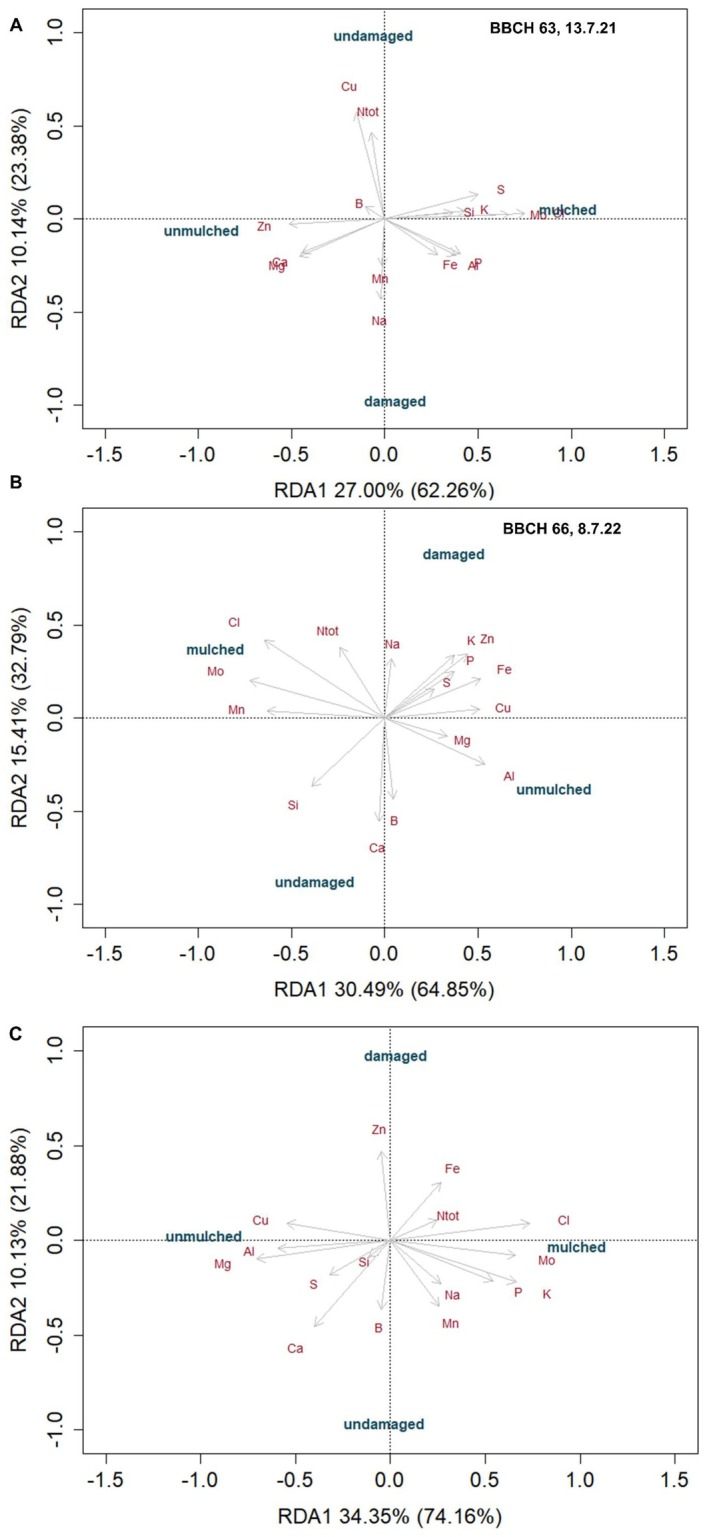
Redundancy analysis for plant sap samples during potato flowering (A) in 2021, (B) 2022, and (C) 2023 originating from unmulched plots and plots with triticale‐vetch mulch application, either highly damaged by CPB or undamaged. See Table S6 for results of statistical analyses.

For the samples taken from mulched and unmulched plants, either damaged or undamaged by CPB in 2021–2023 (2023 with a different potato variety) mulching (*p* = 0.001) and damage level (*p* < 0.05) had a significant effect on the nutrient composition of plant leaves, with no significant interactions in any of the years (Figure 4, Table S6).

The 3 years need to be looked at separately because in 2021, the damage levels of the sampled plants were rather variable (see Section 2). In contrast, in 2022 and 2023, only highly damaged plants were contrasted with undamaged plants. However, in 2023, the potato variety “Anuschka” was sampled, while in the 2 years before “Laura” had been sampled. Nevertheless, some patterns emerged from these samples. As already known from the sampling per plot, higher levels of Cl and Mo were associated with mulched plants, while higher levels of Zn, Cu, Al, and B were associated with unmulched plants (Figure 4). Interestingly, K was associated with mulched plants in 2021 and 2023, when sufficient water was available, but not so in 2022 during the drought.

Nevertheless, summarizing the results of the different sampling years, higher B concentrations were found in undamaged and higher Zn concentrations in damaged leaves; however, the pattern was much weaker in 2021 than in 2022 and 2023. In 2022 and 2023, despite the fact that two different cultivars were sampled, the patterns were quite similar: higher levels of N, Cl, Zn, Fe, and Cu were associated with leaves from plants with very high damage, while higher levels of Ca and B, Mg were associated with undamaged leaves. Again, the behavior of K was different in 2022 when it was associated with damaged leaves, while in the two other years, it was associated with undamaged leaves (Figure 4).

### 3.3 Greenhouse and Feeding Experiments of *L. decemlineata*

**FIGURE 5:**
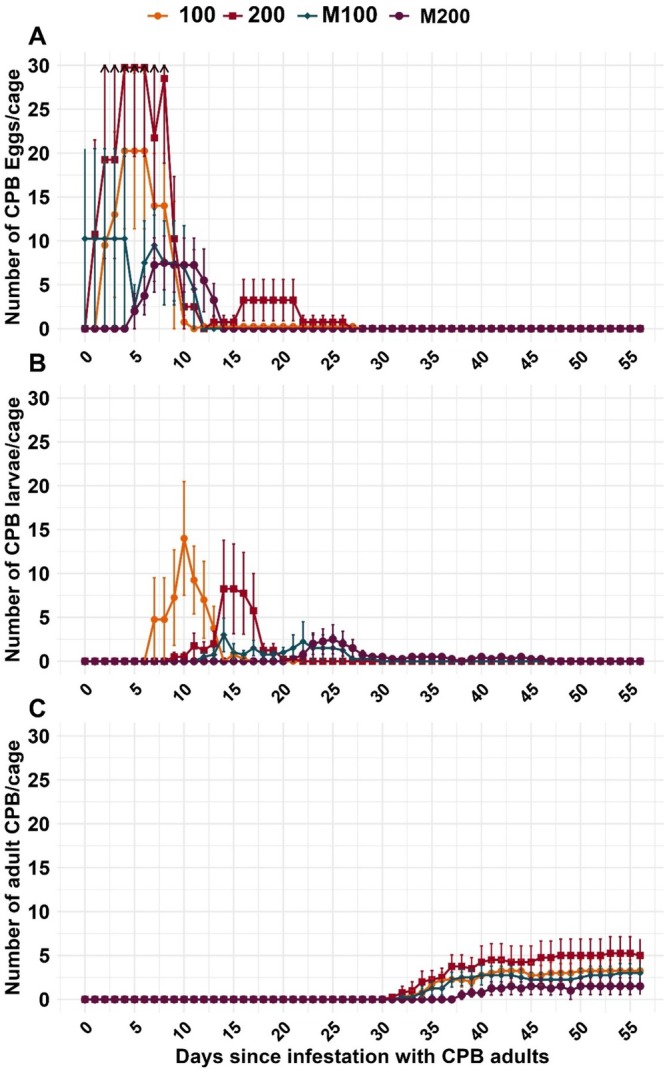
Mean number of Colorado potato beetle individuals in caged mulched (M100, M200) plants and unmulched control plants with hair meal pellet fertilization (100, 200) with standard error grouped by developmental stage ((A) eggs, (B) larvae, and (C) adults) in a greenhouse trial 2021. M100 and 100 were equivalent to 100 kg N/ha and M200 and 200 to 200 kg N/Ha. Black arrows indicate SE exceeding the plot limits. Observation started with the first oviposition and ended with the development to the adult stage. See Table S7 for results of statistical analyses.

**FIGURE 6:**
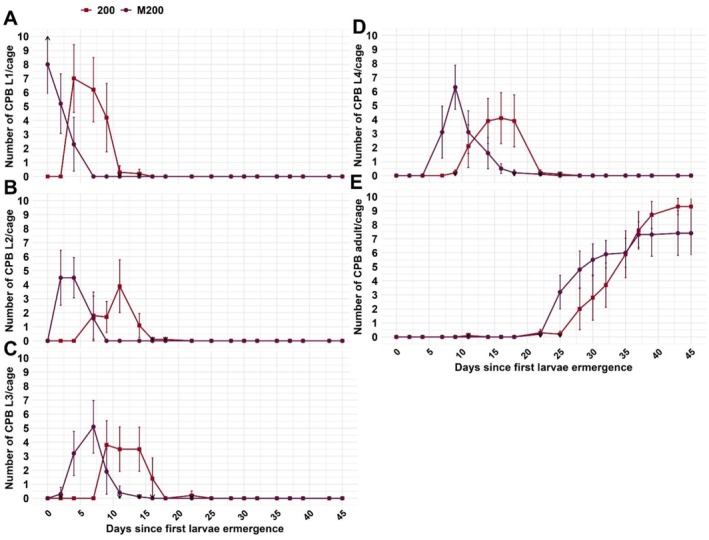
Mean number of Colorado potato beetle (CPB) individuals in caged mulched (M200) plants and unmulched control plants with hair meal pellet fertilization (200) with standard error grouped by developmental stage (A) larval stage 1, B) larval stag 2, C) larval stage 3, D) larval stage 4 and E) adults) in the greenhouse trial 2023. M200 and 200 were equivalent to 200 kg N/Ha. Black arrows indicate SE exceeding the plot limits. Observation started with larval stage 1 and ended with the development to the adult stage.

In the greenhouse experiments in 2021, the total numbers of eggs (*p* = 0.007), larvae (*p* < 0.001), and F1‐adults (*p* < 0.001) were significantly lower in the mulched treatment equivalent to 200 kg N/ha (M200) compared to the unmulched control fertilized with 200 kg N/ha hair meal pellets (200) (Figure 5). Eggs were deposited later and in lower numbers, and the hatching rate was lower in the 200‐treatment cages (41%) than in the M200 ones (60%) (Figure 5, Table 3). As the total survival of eggs was very low in the experiments in 2021 due to limited plant material in only four replicates, the experiment was repeated in 2023, reducing the larvae to 10 individuals with only 1 N‐application rate but 10 replicates per treatment. Of these, 93% survived in the unmulched treatment and 79% in the mulched (9.3 or 7.9 of 10 L1‐larvae reached the adult stage, respectively), with no statistically significant difference. Also, the total duration from larval stage 1 to reaching the adult stage (excluding egg development) did not differ significantly (unmulched 35 and mulched 36 days) (Table 3, Figure 6).

**TABLE 3:** Observation period (developmental stages), mean and SE of hatching rate (%), survival (%), and duration of development (days for observation period) of Colorado potato beetle individuals in caged mulched (M100, M200) plants and unmulched control plants with hair meal pellet fertilization (100, 200) with standard error in greenhouse trials in 2021 and 2023. M100 and 100 were equivalent to 100 kg N/ha and M200 and 200 to 200 kg N/Ha. Observation started with the first oviposition in 2021 and larval stage 1 in 2023 and ended with the complete development to the adult stage.

Year	Treatment	Observation period	Hatching rate (%)		Survival (%)		Duration (days)	
			Mean	SE	Mean	SE	Mean	SE
2021	100	Egg—adult	51.6	18.7	14.5	5.5	43.7	2.0
	200		41.0	17.8	13.7	5.4	44.0	3.6
	M100		71.2	10.5	24.9	12.6	44.7	4.5
	M200		59.7	25.5	18.4	9.2	39.0	2.1
2023	200	Larval stage 1—adult	—	—	93.0	3.7	34.5	0.7
	M200		—	—	79.0	6.6	35.7	1.7

*Note:* M100 and 100 were equivalent to 100 kg N/ha and M200 and 200 to 200 kg N/Ha. Black arrows indicate SE exceeding the plot limits. Observation started with the first oviposition in 2021 and larval stage 1 in 2023 and ended with the complete development to the adult stage.

**FIGURE 7:**
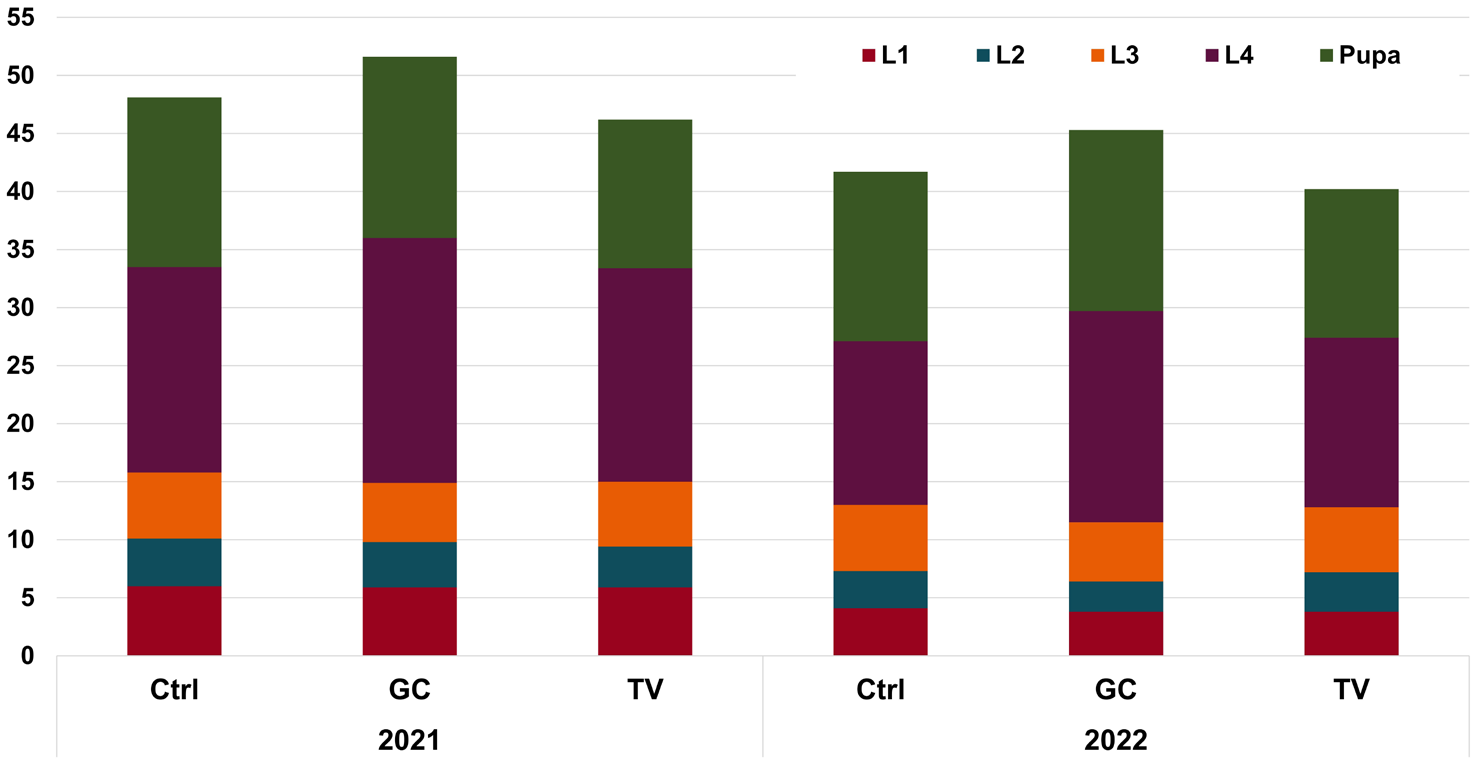
Mean duration in days of CPB developmental stages: Larval stage 1–4, and pupa development until adult stage in feeding experiments in Petri dishes with leaf material from originating plots in the unmulched control (Ctrl) and plots with grass clover (GC) or triticale‐vetch (TV) mulch application. Total duration is calculated based on the sum of the shown stages and does not include egg development. Kruskal–Wallis with Dunn test did not show significant differences. For SE, see Table S8.

In the feeding trial with detached leaves, survival did not differ significantly between treatments. In 2021, 36% of the total number of eggs reached the adult stage in the grass‐clover and 27% in the triticale‐vetch mulch treatment compared to 30% in the unmulched control. When reduced to 10 larvae in 2022, 64% survived up to the adult stage in the grass‐clover and 72% in the triticale‐vetch mulch treatment compared to 69% in the unmulched control (Table 4). Hatching rates were generally above 90% and did not differ between treatments (Table 4). Similarly, the durations of the different developmental stages, from larval stage 1 up to finalizing the pupation and, therefore, reaching the adult stage, did not differ significantly (Figure 7). Stage 4 larvae reached their maximum weight of on average 2.1 g after 12 days (Table 4).

**TABLE 4:** Observation period (developmental stages), hatching rate (%), survival (%), days to maximum weight, and maximum weight of larval stage 4 of Colorado potato beetle individuals in feeding experiments in Petri dishes with leaf material from originating unmulched control (M‐), grass‐clover (GC), and triticale‐vetch (TV) mulch plots in 2021 and 2022.

Year	Mulch	Observation period	Hatching rate (%)		Survival (%)		Days to max. weight		Weight
			Mean	SE	Mean	SE	Mean	SE	Max.
2021	Ctrl	Egg—adult	98.3	2.9	29.5	8.6			
	GC		96.0	1.5	35.6	11.3			
	TV		95.4	2.5	27.3	8.8			
2022	Ctrl	Larval stage 1—adult	—	—	57.9	9.3	12.1	0.7	2.12
	GC		—	—	61.3	5.8	12.3	0.5	2.03
	TV		—	—	64.9	5.9	12.6	0.6	2.25

## 4 Discussion

Our observations revealed intriguing patterns regarding the association of certain nutrients in potato foliage with the application of mulch or high levels of herbivory by CPB versus none. Overall, foliar Mo, Cl, and K contents were repeatedly higher in the mulched treatment, while Mg and, to a lesser degree, Zn contents were repeatedly higher in the absence of mulch. In undamaged leaves, B contents were higher, while Zn contents were higher in damaged leaves. Changes in nutrient contents during 3 weeks from the beginning to the end of flowering were small, with some increases in Ca and B contents and decreases in Zn. These changes are normal and have been generally observed over the potato season (Rowe and Westermann 1993). Despite the associations of N, Cl, Zn, Fe, and Cu with very high CPB damage and Ca, B, and Mg with low damage, none of the correlations between foliar nutrient contents and damage levels by CPB were statistically significant. When excluding potentially interacting effects such as migration, predation, and microclimatic differences that are always present in the field, the effects of mulch on CPB development were inconclusive. There were no discernible effects on CPB development, hatching rate, survival, and weight when fed in Petri dishes with field‐collected leaves from mulched versus unmulched plants, despite the differences in nutrient contents in the two types of leaves. When working with mulched and unmulched caged plants, results differed drastically between experiments. While in the first experiment, beetles laid significantly fewer eggs on mulched plants, this was not repeatable, and no differences in development time could be documented in both experiments.

The lack of effects of plant materials originating from mulched and unmulched field plants on beetle fitness is in contrast to observed differences in the field with uncaged plants (Weiler et al. 2025, n.d.) or with caged plants amended with manure (Alyokhin and Atlihan 2005). This suggests that the nutritional value, which was the differing factor in the feeding experiments, plays only a minor role in the effects found under field conditions in previous studies (Brust 1994; Junge et al. 2022; Weiler et al. 2025, n.d.). This is, insofar interesting as the leaf analysis showed that the composition of the nutrients in the leaflets differed significantly between the different treatments. While reduced egg numbers deposited at a later timepoint were observed in one of our greenhouse experiments, this was not repeatable in the second experiment despite the use of a higher number of replicates and a concentration on the largest differences found in the first experiment.

Except for the first year, when N levels were very low in all treatments, N contents in mulched plants were somewhat lower than in unmulched plants in our experiments. It is not possible to judge if foliar N levels were low, normal, or high, as no data on the foliar N‐contents were previously reported in the studies that had indicated effects of increased N levels on CPB (Jansson and Smilowitz 1985, 1986; Hunt et al. 1992; Boiteau et al. 2008). Generally, our N levels were above the recommended values (Errebhi et al. 1998) with slightly higher values in the unmulched control. Only in 2020, values were optimal, but in 2020, they did not differ between treatments. Therefore, higher N levels do not explain the effect of mulch on CPB reduction.

Despite the known benefits of K for plant defense (Amtmann et al. 2008), K effects were inconclusive. This is likely due to differences in K levels or differences in K availability in the soil. Although all experimental plots had been managed the same way since 2005 and the three experiments had been in the same rotational setting, the soil concentration of K varied between the different experimental fields. Only in 2020, the year when soil concentration was lowest and below the recommended reference, higher K levels were observed in mulched plants. For all experimental fields, an increased K concentration in the soil after mulched potato was observed, highlighting the fertilization potential of transferred organic mulch.

The higher concentration of B in undamaged plants aligns with its established role in enhancing plant defenses against diseases (Soujanya et al. 2021) and in maintaining cell wall integrity through phenolics and lignin content (Hajiboland et al. 2013). However, the lack of differences in B levels between mulched and unmulched plots suggests that it does not explain the reduction effect of mulching on CPB. Furthermore, B does not accumulate in plants as a defense as is known for Zn. While low Zn concentrations can lead to reduced attractiveness to herbivores, potentially also limiting damage, its accumulation as a defense mechanism (Cabot et al. 2019) could explain the higher levels observed in damaged material. Such an accumulation effect due to herbivory might also explain the higher concentrations of Zn found by Alyokhin et al. (2005) in plots with higher CPB damage. The trend of higher Zn concentrations found in the unmulched treatments could also be related to a generally higher leaf damage by CPB that might have triggered Zn accumulation in the leaves (Alyokhin et al. 2005; Soujanya et al. 2021).

Mo and Cl were always higher in the mulched plots. The mulch materials represent a considerable input of Cl, as also documented by the higher increases in Cl contents during the season in mulched plots. For Cl, no beneficial effects on plant health are known (Altieri and Nicholls 2003; Datnoff et al. 2023). Mo is needed for the plant's nitrogen metabolism, but little is known about its effects on plant disease and pests (Roychoudhury and Chakraborty 2022). Mg was mostly higher in the unmulched control. In general, values often exceeded the recommendation, with excess or deficiency potentially increasing plant disease (Tripathi et al. 2022). Excess was observed in all treatments, but higher in the unmulched control. Nevertheless, the effects were not stable enough to explain an effect on CPB dynamics.

A better supply of K, Cl, and Mo by applying transferred organic mulches as fertilizers, in case of deficiencies, can be interesting to improve general plant nutrition (Roychoudhury and Chakraborty 2022) and may also improve resistance to other pests and diseases (Amtmann et al. 2008; Dordas 2008; Tripathi et al. 2022), but they do not seem to be the most relevant factor reducing CPB in mulched potatoes as the effects were not as stable as the reduction of the leaf damage and the CPB numbers (Weiler et al. 2025). In accordance with our previous results from a mark‐release trial, CPB prefers unmulched plants for initial migration and egg deposition (Weiler et al. n.d.).

## 5 Conclusion

The reducing effects of transferred organic mulches on the CPB *L. decemlineata* observed in the field could not be explained by any of the observed changes in foliar nutrient contents in reaction to the application of transferred organic mulch. Feeding CPB with leaf material from mulched plants did not affect developmental time, hatching rate, weight, or survival, while the effects on the number of eggs on caged mulched plants were inconclusive. The “mineral balance hypothesis” as stated by Phelan et al. (1996) and deductively supported by Alyokhin et al. (2005) can neither be supported nor rejected by our results. This hypothesis, in fact, is difficult to study as there is only scant data and suggestions as to what optimal nutrient contents should be in potato foliage, except for some info on the optimal N values for optimum yield (Errebhi et al. 1998). Therefore, we conclude, that initial migration effects due to a physical barrier as documented by Weiler et al. (n.d.) and the effects of mulch on microclimatic conditions in the field Weiler et al. (2025) play a more relevant role in the reduction of CPB through transferred organic mulches than direct effects on the plant nutritional status or nutrient balance. Organic mulch in any case presents a sustainable source of plant nutrients.

## Conflicts of Interest

The authors declare no conflicts of interest.

## Supporting information


Data S1.

## Data Availability

The datasets presented in this study can be found in online repositories. The names of the repository/repositories and accession number(s) can be found below: https://doi.org/10.48662/daks‐84; DaKS—Datarepository Kassel University.
