# Supplementary material for: Mulching Effects on Nutrient Contents of Potato Foliage and Colorado Potato Beetle Fitness
Source: Plant Environ Interact. 2025 Jun 5;6(3):e70059. doi: 10.1002/pei3.70059 (PMC12138577; doi:10.1002/pei3.70059)
Supplement: Supplementary file 1 — Data S1. [file PEI3-6-e70059-s001.docx]

# SUPPORTING INFORMATION


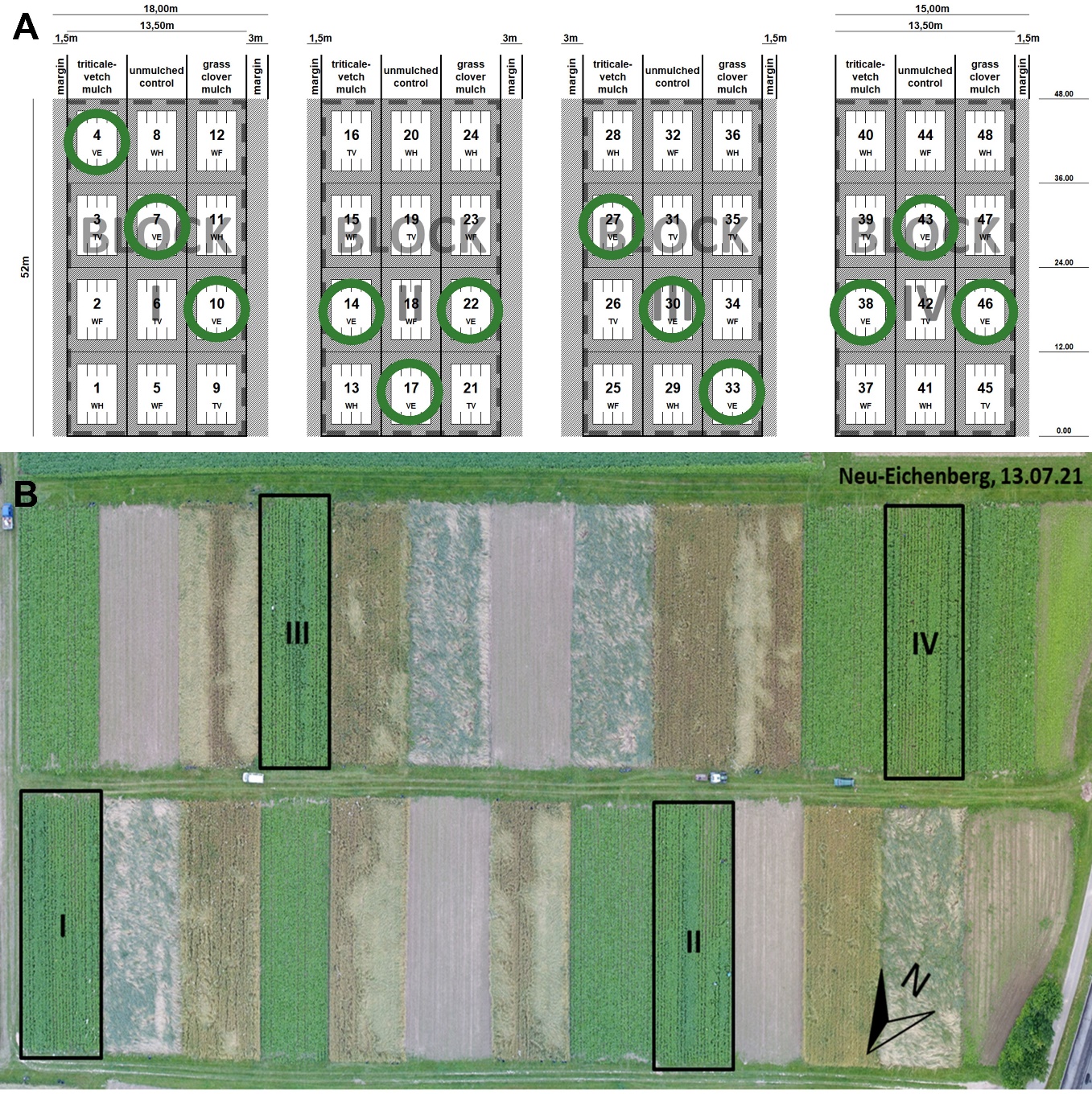


**Figure A 1 Overview of the experimental site in 2021.** The four replicates (roman numerals and boxes) were established within a set of 24 15*50m main plots established in 2005 as four replicates of a six-year rotation. Within each main plot, a split plot design of three 4.5 m wide strips was established. One strip was left unmulched, and received 100 kg N as hair meal pellets as fertilizer, one received triticale-vetch and one grass clover mulch. Three different cover crops and a weedy control preceding potatoes were assigned randomly within each strip. A: Field plan with green circles marking the plots used for the study; WH: winter hardy cover crop mix, TV: Triticale-vetch mix, VE: winter vetch, WF: weedy fallow. Assessed plots are marked with green circles. B: Aerial photo of all 24 plots marking the position of the experimental plots in 2021.

**Table A 1 Field management, CPB sampling dates and developmental stage of potato (BBCH, numbers in brackets)**

| Activities | 2020 | 2021 | 2022 | 2023 |
| --- | --- | --- | --- | --- |
| Cover crop termination, incorporation (4 cm deep) with ferment, fertilization of control plots, seed bed preparation 12 cm deep | 20/21.4. | 30.4. | 20./29.4. | 14./18.5. |
| Potato planting, hilling | 22.4. | 3.5. | 26.4. | 19.5. |
| Mulch application, determine mulch height, mass and N content | 19./20.5. | 9./10.6. | 25.5. | 6.6. |
| Assessment of Leaf damage of CPB | 14.7.(73), 16.7.(79), 20.7.(79), 22.7.(80), 24.7.(81) | 22.6.(27) 26.6.(45) 28.6.(49) 6.7.(59) 8.7.(60) 12.7.(63) 15.7.(66) 19.7.(67) 22.7.(69) 28.7.(70) | 13.6.(24)  8.7.(66)  26.7.(71) | - |
| Plant sap assessment for mulching materials | 24.7. (81) | 7.7. (60), 12.7. (63),  28.7. (70) | 8.7.22 (66),  26/27.7. (70) | 10.7.23 (68) |
| Assessment of plant sap regarding damage |  | 13.7.21 (63) | 8.7.22 (66) | 12.7.23 (68) |
| Harvesting potatoes | 7.9.-10.9. | 8.9.-13.9. | 24./25.9. | 24.9. |

**Table A 2: Details on statistical analysis of on-field assessments** **of plant sap sampling, leaf damage of CPB and controlled CPB experiments.**

| **Assessment** | **Analysis** |
| --- | --- |
| Leaf nutrient composition | ANOVA + Tukey Test for normally distributed data  Kruskal-Wallis Test + Dunn Test for not normally distributed data  Correlation with leaf damage on-field 🡪 no significant interactions found |
| Damaged versus undamaged leaves | Model: rda(formula = Year[, 11:26] + 1 ~ mulch * Damage + Condition(Block), data = VORANII, scale = T, na.action = na.exclude) |
| CPB leaf damage on-field | Calculation of area under the curve (AUC); comparison with Kruskal-Wallis Test + Dunn Test |
| CPB greenhouse experiment | Model: zeroinfl(formula = CPB developmental stage~ Treatment \| Days, data = CPB_I, dist = "negbin") + Tukey Test with emmeans  Survival and hatching rates: Kruskal-Wallis Test + Dunn Test |
| CPB feeding trial | Kruskal-Wallis Test + Dunn Test |

**Table A 3** **Monthly mean temperature and precipitation in Neu-Eichenberg,** North Hesse, for the months of April to July 2020 to 2023 as well as the annual mean temperature and total annual precipitation (right column) and the long-term average (below). The deviations from the long-term average (1991-2020) are colored according to the color scale below.


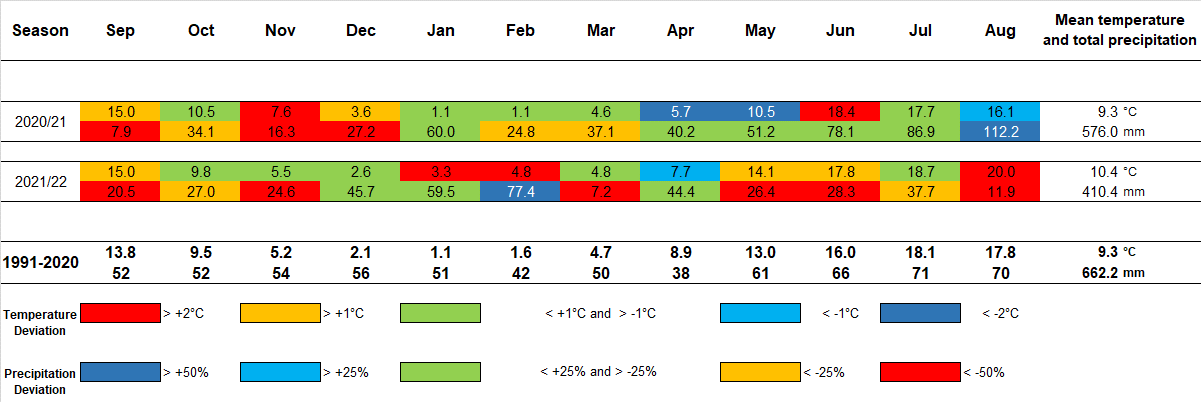


**Table A 4 Recommended reference concentration for micro- and macronutrients for the potato** from personal communication with NovaCropControl in the Netherlands.

| **Nutrient** | **Reference concentration (ppm)** |
| --- | --- |
| N | 1270-1720 |
| P | 90-220 |
| K | 3650-5050 |
| Ca | 625-2050 |
| S | 120-220 |
| Mg | 470-760 |
| Cl | 710-1330 |
| B | 1.6-3.2 |
| Zn | 1.45-2.95 |
| Mn | 7.0-16.9 |
| Mo | 0.05-0.15 |
| Fe | 2.6-4.9 |
| Al | <0.50-1.50 |
| Na | 4.0-10.0 |
| Cu | 0.35-0.85 |

**Table A 5 Contents of calcium-acetate-lactate (CAL) extractable P, K, and Mg** for unmulched control plots (Ctrl), plots with grass-clover (GC) and triticale-vetch (TV) mulch application before and after potato for the experimental seasons 2020, 2021 and 2022. Sampling date is indicated.

| **Mulch** | **Before potato** | | | | **After potato** | | | |
| --- | --- | --- | --- | --- | --- | --- | --- | --- |
|  | **Date** | **P** | **K** | **Mg** | **Date** | **P** | **K** | **Mg** |
| Ctrl |  |  |  |  | 31.03.21 | 5.5 | 9.0 | 13.1 |
| GC |  |  |  |  |  | 5.5 | 13.0 | 13.0 |
| TV |  |  |  |  |  | 6.3 | 10.5 | 12.4 |
| Ctrl | 30.03.21 | 10.0 | 12.8 | 13.5 | 23.11.21 | 18.5 | 23.5 | 14.2 |
| GC |  | 12.3 | 12.8 | 14.3 |  | 23.8 | 28.5 | 14.7 |
| TV |  | 10.0 | 10.3 | 14.0 |  | 20.5 | 27.8 | 14.8 |
| Ctrl | 29.03.22 | 16.8 | 17.3 | 12.8 | 12.06.23 | 25.8 | 25.1 | 14.5 |
| GC |  | 15.0 | 16.0 | 12.4 |  | 24.1 | 36.3 | 14.8 |
| TV |  | 17.0 | 18.3 | 12.9 |  | 27.5 | 32.4 | 15.1 |

**Table A 6 Statistical results of redundancy analysis for plant sap samples** during potato flowering originating from unmulched plots and plots with triticale-vetch mulch application either highly damaged by CPB or undamaged.

| **Year** | **Treatment** | **Df** | **Variance** | **F** | **p-value** |
| --- | --- | --- | --- | --- | --- |
| **2021** | mulch | 1 | 4.31 | 7.99 | 0.001 *** |
|  | Damage | 1 | 1.63 | 3.02 | 0.015 * |
|  | mulch:Damage | 1 | 1.00 | 1.85 | 0.110 |
|  | Residual | 9 | 4.85 |  |  |
| **2022** | mulch | 1 | 4.52 | 8.58 | 0.001 *** |
|  | Damage | 1 | 2.68 | 5.08 | 0.003 ** |
|  | mulch:Damage | 1 | 0.32 | 0.61 | 0.67 |
|  | Residual | 9 | 4.75 |  |  |
| **2023** | mulch | 1 | 5.49 | 9.52 | 0.001 *** |
|  | Damage | 1 | 1.59 | 2.76 | 0.039 * |
|  | mulch:Damage | 1 | 0.32 | 0.56 | 0.70 |
|  | Residual | 9 | 5.19 |  |  |

**Table A 7 Statistical results of number of Colorado potato beetle individuals in caged mulched (M100, M200) plants and unmulched control plants with hair meal pellet fertilization (100, 200)** grouped by developmental stage (eggs, larvae, adults) in a greenhouse trial 2021. M100 and 100 were equivalent to 100 kg N/ha and M200 and 200 to 200 kg N/Ha Black arrows indicate SE exceeding the plot limits. Observation started with first oviposition and ended with the development to the adult stage.

| **Stage** | **contrast** | **estimate** | **SE** | **df** | **z.ratio** | **p.value** |
| --- | --- | --- | --- | --- | --- | --- |
| **Eggs** | (100) - (200) | -1.05283882 | 0.57417063 | Inf | -1.8336689 | 0.2574447 |
|  | (100) - (M100) | -0.89961859 | 0.76109334 | Inf | -1.18200823 | 0.63824756 |
|  | (100) - (M200) | 0.71999708 | 0.42191273 | Inf | 1.70650711 | 0.32019053 |
|  | (200) - (M100) | 0.15322024 | 0.83260925 | Inf | 0.18402418 | 0.99778985 |
|  | (200) - (M200) | 1.7728359 | 0.55115599 | Inf | 3.21657742 | 0.00710304 |
|  | (M100) - (M200) | 1.61961567 | 0.73520358 | Inf | 2.20294855 | 0.12237774 |
| **Larvae** | (100) - (200) | 0.00895268 | 0.55747007 | Inf | 0.01605949 | 0.99999851 |
|  | (100) - (M100) | 0.78495697 | 0.49023155 | Inf | 1.60119636 | 0.37788776 |
|  | (100) - (M200) | 2.17234186 | 0.40890207 | Inf | 5.31262128 | 6.463E-07 |
|  | (200) - (M100) | 0.77600429 | 0.50471427 | Inf | 1.53751208 | 0.41495936 |
|  | (200) - (M200) | 2.16338918 | 0.42483718 | Inf | 5.09227838 | 2.1125E-06 |
|  | (M100) - (M200) | 1.38738489 | 0.32954591 | Inf | 4.20998974 | 0.00014991 |
| **Adults** | (100) - (200) | -0.58540482 | 0.10821903 | Inf | -5.40944446 | 3.7833E-07 |
|  | (100) - (M100) | 0.11965313 | 0.09304704 | Inf | 1.28594235 | 0.57189801 |
|  | (100) - (M200) | 0.46167924 | 0.10831 | Inf | 4.26257252 | 0.00011884 |
|  | (200) - (M100) | 0.70505796 | 0.10851215 | Inf | 6.49750228 | 4.8989E-10 |
|  | (200) - (M200) | 1.04708406 | 0.12891631 | Inf | 8.12219982 | 5.1736E-14 |
|  | (M100) - (M200) | 0.34202611 | 0.10495 | Inf | 3.25894347 | 0.00615319 |

**Table A 8** **Mean duration with SE in days of CPB developmental stages: larval stage 1-4, and pupa development** until adult stage in feeding experiments in petri dishes with leaf material from originating unmulched control (M-), grass-clover (GC) and triticale-vetch (TV) mulch plots in 2021 and 2022. Total duration is calculated based on the sum of the shown stages and does not include egg development. Kruskal-Wallis with Dunn test did not show significant differences.

| **Year** | **Mulch** | **L1** | | **L2** | | **L3** | | **L4** | | **Pupa** | |
| --- | --- | --- | --- | --- | --- | --- | --- | --- | --- | --- | --- |
|  |  | **Mean** | **SE** | **Mean** | **SE** | **Mean** | **SE** | **Mean** | **SE** | **Mean** | **SE** |
| 2021 | M- | 6.0 | 0.4 | 4.1 | 0.4 | 5.7 | 0.7 | 17.7 | 2.0 | 14.6 | 1.0 |
|  | GC | 5.9 | 0.5 | 3.9 | 0.6 | 5.1 | 1.0 | 21.1 | 1.7 | 15.6 | 1.1 |
|  | TV | 5.9 | 0.5 | 3.5 | 0.5 | 5.6 | 1.3 | 18.4 | 1.7 | 12.8 | 1.3 |
| 2022 | M- | 4.1 | 0.4 | 3.2 | 0.7 | 5.7 | 0.7 | 14.1 | 1.5 | 14.6 | 1.0 |
|  | GC | 3.8 | 0.5 | 2.6 | 0.5 | 5.1 | 1.0 | 18.2 | 1.8 | 15.6 | 1.1 |
|  | TV | 3.8 | 0.4 | 3.4 | 0.5 | 5.6 | 1.3 | 14.6 | 1.3 | 12.8 | 1.3 |
